# Supplementary material for: Iron-based trinuclear metal-organic nanostructures on a surface with local charge accumulation
Source: Nat Commun. 2018 Aug 10;9:3211. doi: 10.1038/s41467-018-05543-4 (PMC6086834; doi:10.1038/s41467-018-05543-4)
Supplement: Supplementary file 1 — Supplementary Information [file 41467_2018_5543_MOESM1_ESM.pdf]

Iron-based trinuclear metal-organic nanostructures on a surface with local charge accumulation

Krull et al.

Iron-based trinuclear metal-organic nanostructures on a surface with local charge accumulation

Supplementary Information

Cornelius Krull[1], Marina Castelli,[1,2], Prokop Hapala[3], Dhaneesh Kumar[1,2,4], Anton Tadich[5], Martina Capsoni[6], Mark T. Edmonds[1,2,4], Jack Hellerstedt[1,2,3], Sarah A. Burke[6,7,8], Pavel Jelinek[3,9] and Agustin Schiffrin[1,2,4*]*

[1]School of Physics & Astronomy, Monash University, 19 Rainforest Walk, Clayton 3800, Australia

[2]Monash Centre for Atomically Thin Materials, Monash University, 20 Research Way, Clayton 3800 Australia

[3]Institute of Physics of the CAS, Cukrovarnicka 10, Prague 16200, Czech Republic

[4]ARC Centre of Excellence in Future Low-Energy Electronics Technologies, Monash University, 19 Rainforest Walk, Clayton 3800, Australia

[5]Australian Synchrotron, 800 Blackburn Road, Clayton, Victoria 3168, Australia

[6]Department of Physics and Astronomy, University of British Columbia, 6224 Agricultural Road, Vancouver, British Columbia, Canada V6T 1Z1

[7]Department of Chemistry, University of British Columbia, 2036 Main Mall, Vancouver, British Columbia, Canada V6T 1Z1

[8]Stewart Blusson Quantum Matter Institute, University of British Columbia, 2355 East Mall, Vancouver, British Columbia, Canada V6T 1Z4

[9]RCPTM, Palacky University, Šlechtitelů 27, 783 71, Olomouc, Czech Republic

*Corresponding author: agustin.schiffrin@monash.edu

*Corresponding author: jelinekp@fzu.cz

Contents

Supplementary Note 1: Interaction between carbon monoxide and metal-organic complex 2
Supplementary Note 2: NcAFM experiments 3
  Influence of tip-sample distance 3
  ncAFM image filtering process 5
  Adsorption geometry from ncAFM imaging 6
  Vertical ncAFM imaging 8
Supplementary Note 3: (dI/dV)/(I/V) STS of metalated TPPT species 9
Supplementary Note 4: Nature of the ncAFM central feature of the coordination node 10
Supplementary Note 5: Comparison with hypothetical di-iron coordination node 11
Supplementary Note 6: NEXAFS spectroscopy, Bader analysis and oxidation state of iron atoms 13
Supplementary Note 7: Van der Waals parameters for AFM simulations 15
Supplementary Note 8: Chain adsorption geometry 16
Supplementary References 17

Supplementary Note 1: Interaction between carbon monoxide and metal-organic complex

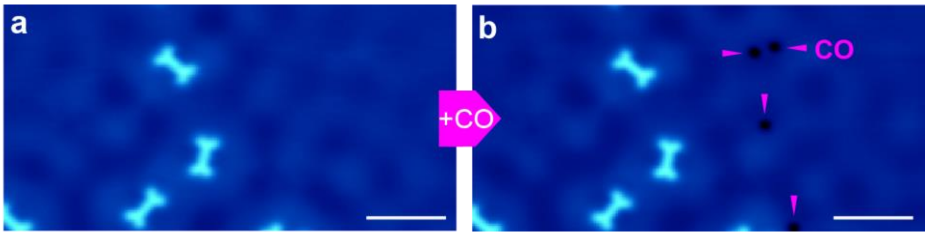

Supplementary Figure 1. Constant-current STM topography image of TPPT/Ag111 (I_t = 25 pA, V_b = 20 mV) with a Pt/Ir tip, before (a) and after (b) dosing of carbon monoxide (CO). CO molecules are imaged as depressions, indicated with fuchsia arrows. Scale bars: 10 nm.

Metal-organic complexes can be reactive to small molecules such as carbon monoxide (CO).[1] CO molecules introduced in the STM chamber – for CO-tip STM and ncAFM imaging (see main text) – can hence interact with the self-assembled nanostructures studied, even at extremely low coverages. To address this, we closely monitored the appearance of any new species that could potentially be related to interactions with CO, both for pristine and metalated TPPT. Supplementary Figure 1 shows an STM topography image of pristine TPPT on Ag(111), before and after dosing CO. After dosing, small depressions appear on the Ag (fuchsia ticks in Supplementary Fig. 1b). These features are due to individual isolated CO molecules.[2]

For Fe metalated TPPT molecules, we observed a new species that only appears after dosing CO. Supplementary Figure 2a shows an STM image (Pt/Ir tip) of a TPPT molecule with each of its *tpy* groups coordinated with an Fe atom, after dosing with CO. Each *tpy-Fe* group is imaged as three protrusions, as opposed to the typical “hammer” shape of a non-decorated *tpy-Fe* (Fig. 3 in main text). By applying a bias voltage pulse close to one of the affected *tpy-Fe* groups in Supplementary Fig. 1a (white cross), we could convert it to the “hammer”-shaped *tpy-Fe* (Supplementary Fig. 2b). Moreover, the STM image after the pulse shows a new depression adjacent to the TPPT. To confirm that this depression is a CO molecule, we used our pick-up procedure to functionalise the STM tip (Methods), and subsequently imaged the same molecule. The resulting image in Supplementary Fig. 2c shows the hallmarks of a CO-functionalised STM tip (Fig. 2 of main text). We thus conclude that the STM imaging of Fe-TPPT-Fe in Supplementary Fig. 2a is the result of *tpy-Fe* groups interacting and binding with CO molecules.

Correspondingly, we also investigated interactions between CO and coordination nodes in the metal-organic nanochains (MOCs). Supplementary Figure 2d shows an STM topography map of a MOC node (circled in grey), imaged differently (asymmetric) than the node discussed in the main text (symmetric). By lateral STM manipulation (Methods), we *deconstructed* this type of node. The STM imaging in Supplementary Fig. 2e reveals that it is composed of a pristine *tpy-Fe* group and a CO-decorated *tpy-Fe* (Supplementary Figs. 2e, f; compare with Supplementary Figs. 2a, b). We unequivocally identify these species by identical STM imaging, for a series of different bias voltages (V_b = -500 mV to 500 mV, data not shown). This type of asymmetric node is hence composed of two *tpy-Fe* groups bridged by a CO ligand. Our ability to identify CO-decorated species allows for the systematic investigation of pristine TPPTs, metalated TPPTs and MOCs. The results reported in the main text correspond to the latter, that is, systems that do not contain CO molecules.

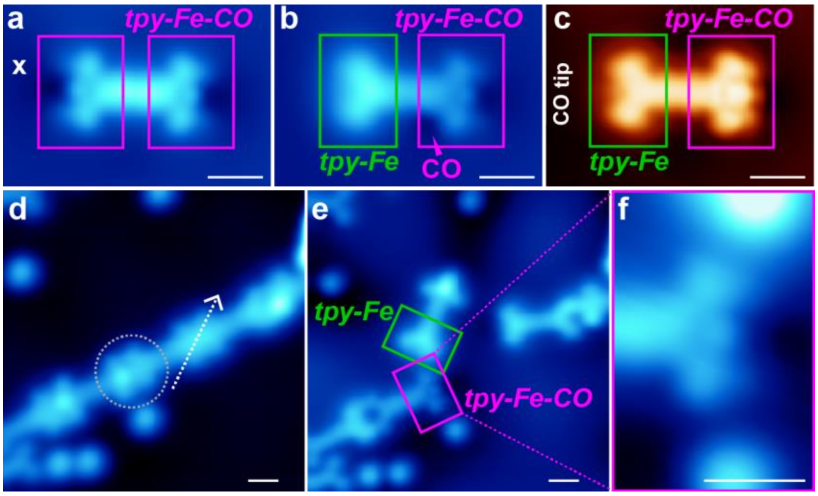

Supplementary Figure 2. Interaction between CO and Fe-TPPT complexes. a, STM image of TPPT molecule with both *tpy-Fe* groups decorated with CO (I_t = 50 pA, V_b = -25 mV). **b,** STM image of the same molecule after applying a bias voltage pulse (V_{pulse}= 2 V, t_{pulse}= 200 ms, I_t = 50 pA, V_b = -25 mV) at the white cross: the CO molecule *jumped* next to one of the distal *pyr* rings, resulting in a pristine left *tpy-Fe* (I_t = 50 pA, V_b = -25 mV). **c,** STM image of molecule in (b), after picking up the adjacent CO molecule with the tip (I_t = 50 pA, V_b = -25 mV). **d,** STM image of MOC. Dashed white arrow indicates tip displacement direction during lateral STM manipulation across symmetric, non-decorated (I_t = 400 pA, V_b = -500 mV). **e,** STM image of *deconstructed* MOC: the node circled in (d) consists of a CO-decorated *tpy-Fe-CO* and a *tpy-Fe* (I_t = 400 pA, V_b = -20 mV). **f,** Zoomed-in image of *tpy-Fe-CO* in (e)**.** STM topography images were recorded with a Pt/Ir tip in (a), (b), (d)-(f) (blue colour coding) and with a CO-functionalised tip in (c) (brown colour coding). Scale bars: 1 nm.

Supplementary Note 2: NcAFM experiments

Influence of tip-sample distance

Non-contact AFM imaging (Fig. 2 of main text) was performed at a constant height of 0.8 Å above a reference height defined by the STM tunnelling setpoint on bare Ag(111): I_t = 25 pA, V_b = 20 mV. Using $I(z)$ measurements we estimated an absolute tip height of 6 ± 1 Å [standard deviation (s.d.)] above the sample.

In addition, we performed ncAFM imaging for varying tip heights ranging from 1.4 to 0.6 Å above the same STM setpoint I_t = 25 pA, V_b = 20 mV (Supplementary Fig. 3). As the tip approaches the molecule, short range repulsive forces become stronger and contribute significantly to the imaging, increasing the intramolecular chemical bond contrast (Supplementary Fig. 3m).[3, 4] At small distances (0.4 Å; 0.6 Å; 0.8 Å), the *pyr* rings of the molecule exhibit a repulsive feature at their centre, which has been observed for other aromatic molecules and is attributed to bending of the CO molecule on the tip.[5] At 0.4 Å above the setpoint (Supplementary Figs. 3k, l), both the *tpy* and *tpy-Fe* groups display these repulsive features. However, their appearance differs significantly, highlighting the effect of the metalation on the *tpy* group; the pristine *tpy* shows this repulsive feature mainly at the centre of the distal *pyr* rings (blue ticks), while the metalated *tpy-Fe* shows the strongest feature at the axial *pyr* (green tick). The *tpy* involved in the coordination node shows the same characteristics as the *tpy-Fe* (red ticks), consistent with our claim that the configurations of the node-*tpy* is identical to that of an unchained *tpy-Fe*. The exact symmetry of such tip bending effects depends strongly on the nanoscale morphology of the CO-tip apex.[5] Consequently, we only compare images acquired with the same functionalised CO tip.

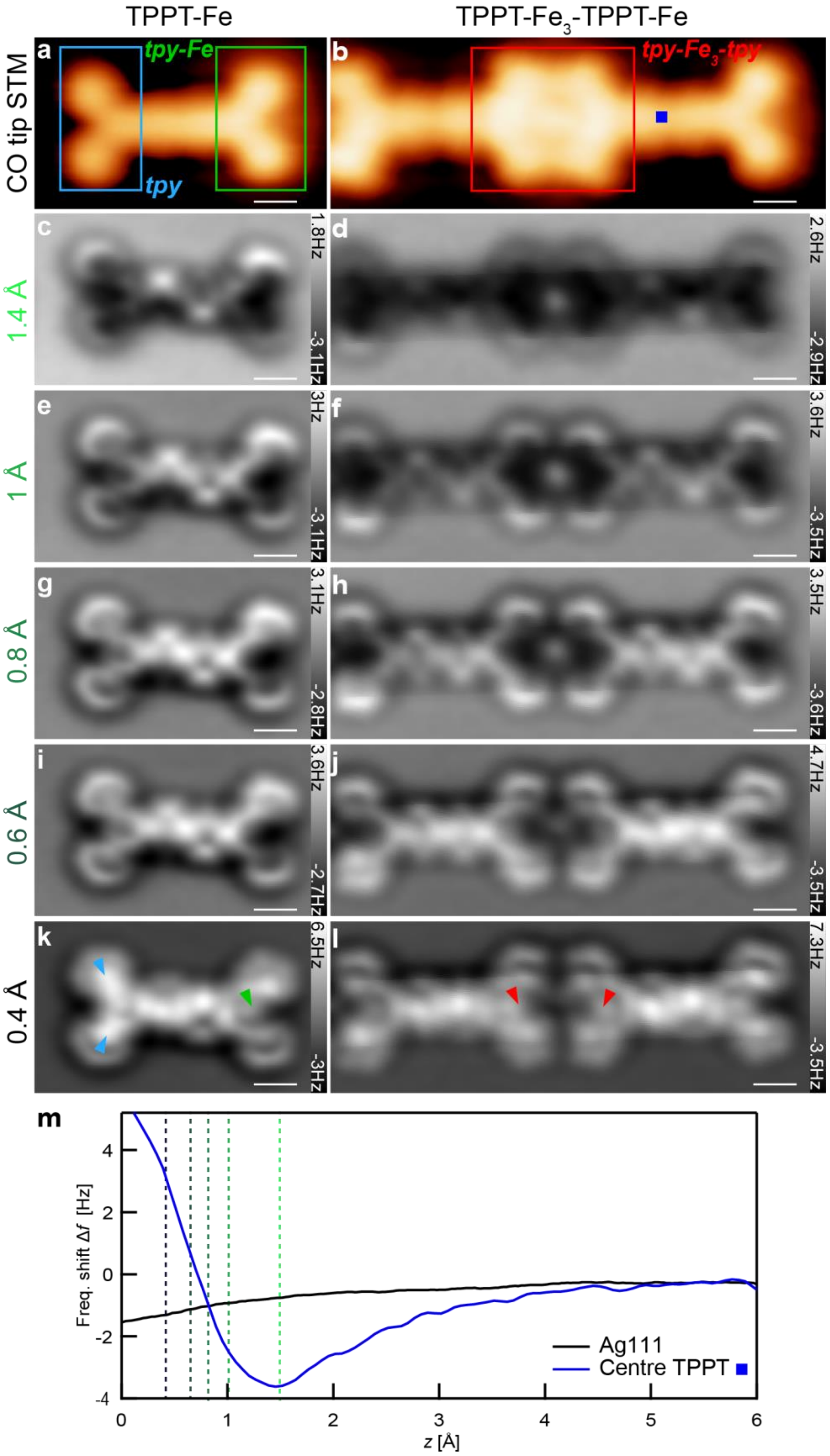

Supplementary Figure 3. CO-tip ncAFM imaging for different tip-sample distances. a, STM topography of a singly metalated TPPT and **b,** of a coordination node in a MOC (I_t = 10 pA, V_b = 20 mV). **c-l,** Constant-height ncAFM images of the same systems at varying tip heights. Gaussian filter applied. Effects due to CO-tip bending are indicated with ticks in (k) and **(**l). **m,** Frequency shift Δf as a function of tip-sample distance z measured at the centre of a TPPT molecule in a MOC [blue square in (a)] and on bare Ag(111). All data were acquired with the *same* CO-functionalised Pt/Ir tip on a qPlus sensor**;** z = 0 refers to a tip height defined by an STM setpoint on bare Ag(111) (I_t = 25 pA, V_b = 20 mV). Scale bars: 5 Å.

ncAFM image filtering process

To enhance the contrast in the ncAFM images, especially in regions with low contrast, e.g., *tpy* groups, we applied a Laplace edge detection filter based on Ref. 6. Supplementary Figure 4 displays the steps of the filtering process for the pristine TPPT, the singly metalated TPPT and the coordination node (note that these images were recorded with a resolution of 128 pixels/nm):

i. Gaussian smoothing with full width at half maximum ~1-2 pixels (multiple times);
ii. Second order edge detection by convolution with a Laplacian edge detection kernel -[0 ¼ 0; ¼ -1 ¼; 0 ¼ 0];
iii. Minimum filter (for sharpening), that is, selection for each pixel of a local minimum within a disk area (we used the Matlab function '*imerode'* with a disk radius of 3 pixels).

The apparent height of the prominent repulsive feature at the centre of the coordination node is affected by the filtering. Supplementary Figure 5 shows the frequency shift Δf measured across and along the coordination node, for each step of the filtering process. For example, the raw ncAFM data show a value of Δf at the centre of the node, which is smaller than that for bare Ag(111) as well as that for the bonds of the adjacent, distal *pyr*'s. The Laplacian filter, which is sensitive to the local

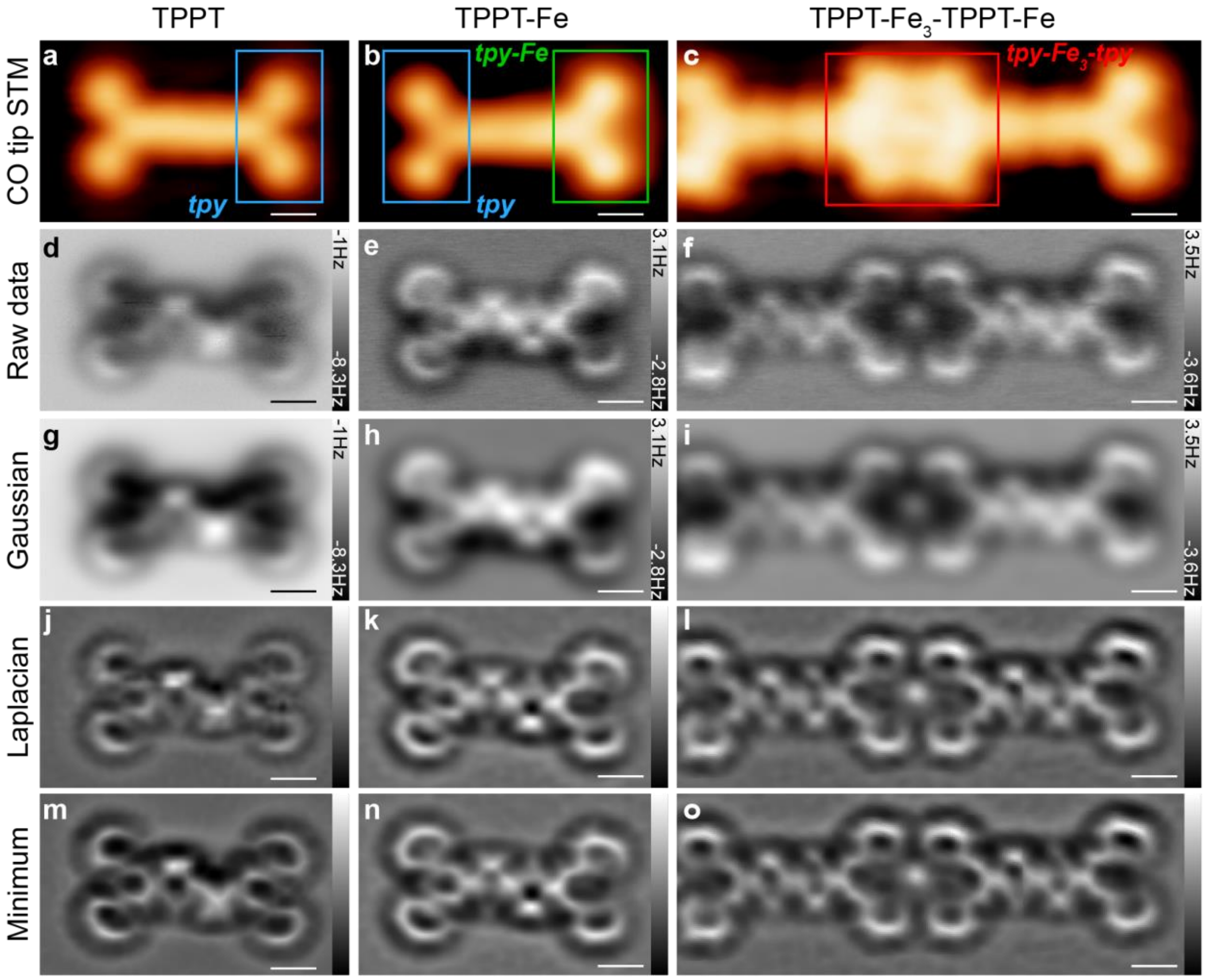

Supplementary Figure 4. NcAFM imaging filtering steps. a-c, STM topography images with CO-functionalised Pt/Ir tip on qPlus sensor (a,b: I_t = 5 pA, V_b = 20 mV; c: I_t = 10 pA, V_b = 20 mV). **d- f,** Raw constant-height ncAFM images, 0.8 Å above an STM setpoint on bare Ag(111) (I_t = 25 pA, V_b = 20 mV). **g-i,** Same images with Gaussian smoothing. **j-l,** Second order Laplacian edge detection. **m-o,** Sharpening using a minimum filter (64 pixels). Scale bars: 5 Å.

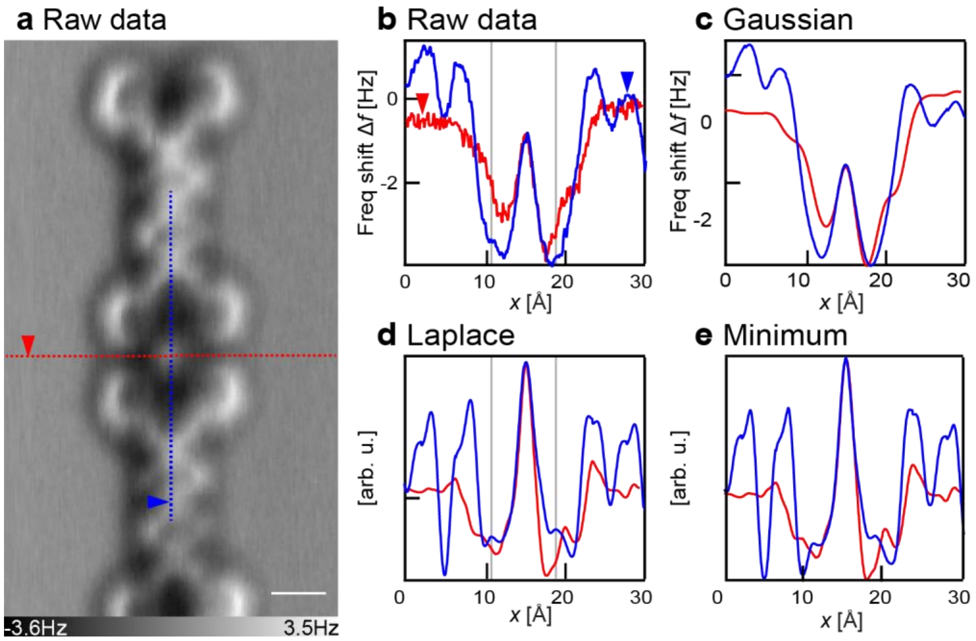

Supplementary Figure 5. Influence of the ncAFM data filtering on the imaging of the coordination node. **a,** Raw constant-height ncAFM data with a CO-functionalised Pt/Ir tip on a qPlus sensor, 0.8 Å above an STM setpoint on the bare Ag(111) (I_t = 25 pA, V_b = 20 mV). **b-e,** NcAFM apparent height profiles across (red) and along (blue) the node, for different steps of the filtering process. Scale bar: 5 Å. Vertical grey lines in (b) and (d) indicate the positions of weak features in the raw data that become emphasized by the Laplace-filtering.

variation of Δf, enhances contrast within the node and renders the apparent height of the central feature larger than the Ag(111) level. In general, the Laplace-filtered images reproduce the topographic features and increase the contrast by emphasizing edges and peaks. It is important to note that the position of features (e.g., peaks) in the original raw image is not altered by Laplace-filtering. For example, the ncAFM height profile across the tri-iron node along the molecular axis (blue curve in Supplementary Fig. 5b) shows five prominent peaks. The heights of these peaks become larger by Laplace-filtering, although the peak positions are not changed (blue curve in Supplementary Fig. 5d). Because of this contrast enhancement, weaker features, present in the raw data, can be emphasized and can become more apparent by Laplace-filtering (e.g., see grey lines in Supplementary Figs. 5b, d). It is important to note that this filtering process does not alter our interpretation of the data.

Adsorption geometry from ncAFM imaging

The ncAFM maps of the TPPT reveal a non-planar adsorption geometry of the molecule (Supplementary Fig. 6), an observation that is confirmed by our DFT calculations. Several molecular moieties are affected: the central phenyl (*ph*) rings (red, α) and the distal *pyr* rings (blue, β) are rotated out of the molecular plane, while the distal *pyr* change their in-plane angle as well (green, γ). To gauge the impact of metalation and chain formation on the molecular conformation, we estimate these angles from the ncAFM data and compare them to the relaxed DFT geometry, for TPPT in various stages of metalation.

We estimate the out-of-molecular-plane angles within the assumption that a given frequency shift over a C-C bond corresponds to a determined height of the tip over this bond. Based on the ncAFM maps recorded at different tip heights above the molecule (Supplementary Fig. 3), we determine the apparent height difference between various points on the moiety (in Supplementary Fig. 6, red arrows on *ph*, and blue arrows on *pyr*), which can then be fitted to a rotated plane. The

finite height resolution of our ncAFM maps (10 pm) contributes to the measurement error. Note that the estimation of the *pyr* rotation is more challenging, since the interaction with the tip is different for nitrogen than for carbon, resulting in an uneven *pyr* plane. To address this, we determined the *pyr* out-of-plane angle by fitting a plane defined by four points on the *pyr* ring away from the nitrogen (blue and cyan arrows in Supplementary Fig. 6).

The in-plane angle γ of the distal *pyr*'s was directly extracted from the ncAFM maps using three anchor points: the C-C bond between *ph* and the axial *pyr* (repulsive maximum indicated by green arrow in Supplementary Fig. 6b); the opposing C atom on each of the distal *pyr*'s (kink in the distal *pyr*; green arrows in Supplementary Fig. 6b).

	ncAFM			DFT		
**TPPT**	pristine	metalated	chain	pristine	metalated	chain
**phenyl rotation (α)**	3 ± 3°	5 ± 2°	2 ± 2°	2.5°	1.9°	1.2°
**pyridine out-of-plane (β)**	3 ± 2°	4 ± 2°	6 ± 2°	1.5°	9.1°	3.3° 4.3°
**pyridine in-plane (γ)**	87 ± 8°	73 ± 6°	80 ± 9°	95.6°	72°	74°

Supplementary Table 1. Molecular conformation changes upon adsorption, metalation, and chain formation. Angles defined as in Supplementary Fig. 6. Errors correspond to twice the standard deviation.

Supplementary Table 1 shows the extracted angles for TPPT in different stages of metalation: pristine TPPT, TPPT metalated on one side and for TPPT in a MOC. We found that these experimental values are in very good agreement with the DFT geometries, for all systems. The out-of-plane rotation of the aromatic moieties is slightly underrepresented in DFT due to an overestimation of the delocalization of π electrons, leading to flatter aromatic systems.[7] Note that the DFT out-of-plane angles (β) for the distal *pyr*'s in the coordination node are slightly asymmetric for the left and right *tpy*. The reduction of the in-plane angle (γ) of the distal *pyr* by 14 ± 5° (s.d.) due to Fe metalation is expected and is consistent with values found in literature for other *tpy-Fe* systems.[8]

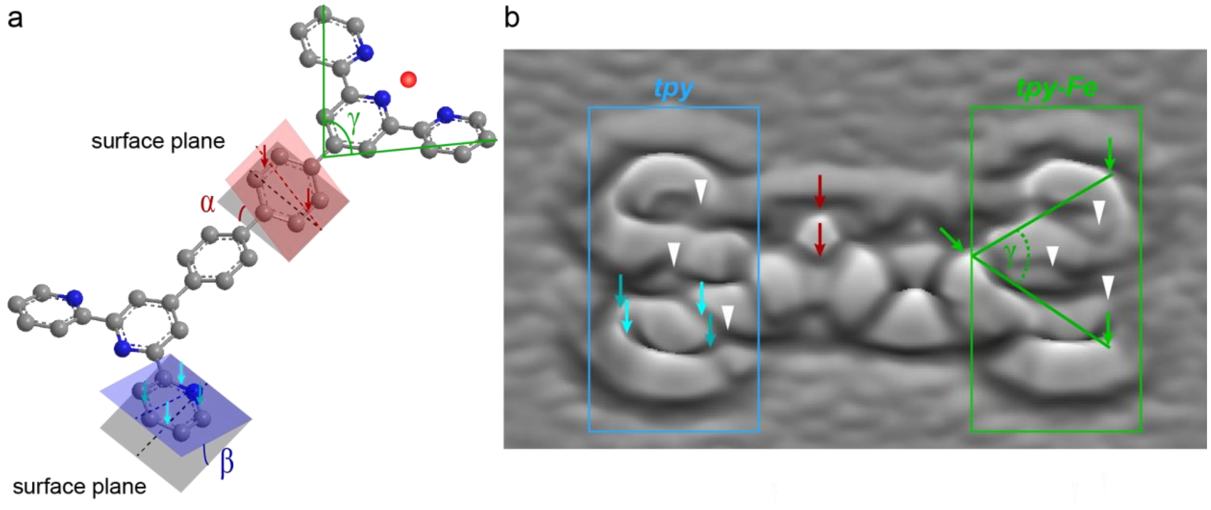

Supplementary Figure 6. TPPT adsorption geometry from ncAFM imaging. a, Structure of singly metalated TPPT. Arrows indicate the position where height values were extracted. **b,** 3D representation of CO-tip ncAFM image of singly metalated TPPT (Supplementary Fig. 4h). White ticks indicate the position of the nitrogen atoms in the *pyr* rings.

Vertical ncAFM imaging

To gain insight into the height dependence of the CO-tip ncAFM central bright feature in the coordination node, we performed vertical ncAFM imaging.[9, 10] This approach consists of acquiring z-dependent frequency shift Δf (z) curves along regions of interest. The Δf (z) acquisition was cut off once Δf reached a fixed minimum or maximum parameter, to avoid strong forces. Vertical ncAFM imaging has been shown to resolve subtle intramolecular conformations of aromatic complexes.[9] Supplementary Figure 7b shows Δf (z) curves taken at different positions x along the tri-iron coordination node in Fig. 2c of the main text [z = 0 corresponds to an STM set point on bare Ag(111), I_t = 5 pA, V_b = 20 mV, which we estimate by $I(z)$ measurements to be 7 ± 1 Å (s.d.) above the Ag(111) surface plane]. The vertical ncAFM imaging along the node shows a shoulder (white ticks in Supplementary Fig. 7b), which we associate with the axial *pyr* groups of the node, indicative of the *pyr* ring bending towards the surface due to the coordination with the Fe adatoms.

A central repulsive feature, in between the two shoulders, is clearly visible above the molecular plane (for $z \sim 0$). This feature was not observed for *tpy*-Fe groups that are not incorporated in a chain, and is a characteristic signature of the tri-iron coordination motif. The simulated vertical frequency shift map across the coordination node exhibits the shoulders associated with the metalation of the *tpy* group (Supplementary Fig. 7c, white ticks). Importantly, this simulation (which includes electrostatic interactions between the sample and the charged CO molecule on the tip; see Supplementary Fig. 9 also) reproduces our z-dependent experimental ncAFM data, in particular the bright central feature above the molecular plane. At this distance, electrostatic forces are the main contribution to ncAFM contrast[3], indicating an electrostatic origin of the feature.

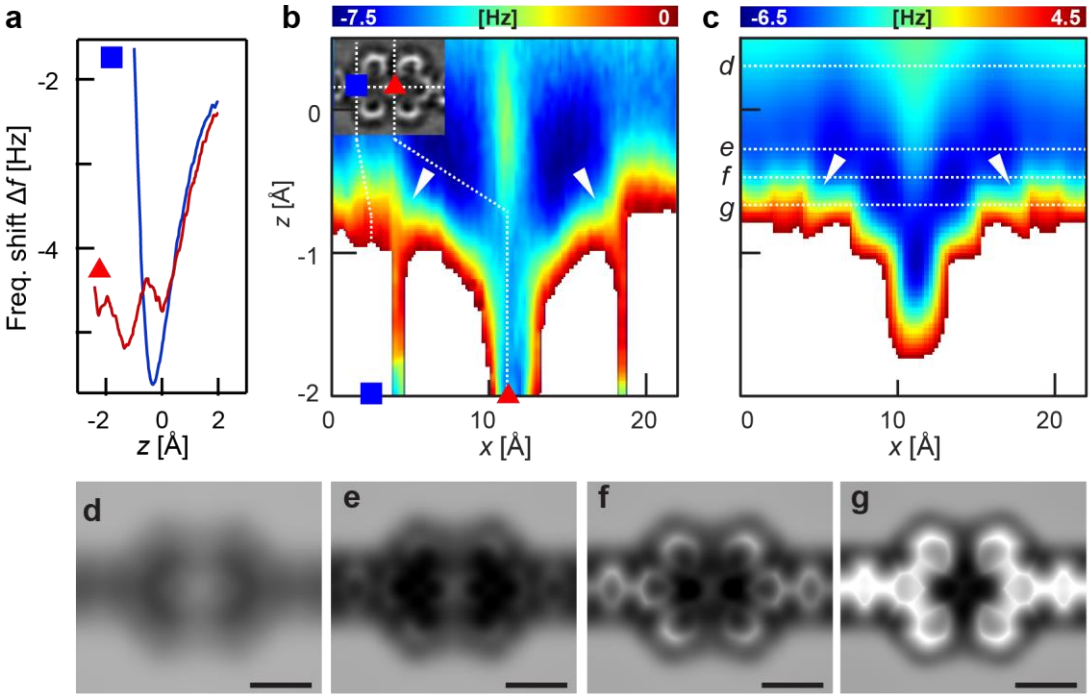

Supplementary Figure 7. Height dependence of CO-tip ncAFM along the Fe-TPPT coordination node. **a,** Frequency shift Δf as a function of tip height z, on TPPT in MOC (blue) and at centre of coordination node. **b,** Vertical ncAFM map across a chain node. White ticks indicate the shoulder associated with the axial metalated *pyr* group. The z position is defined with respect to an STM setpoint on a bare patch of Ag(111) (I_t = 5 pA, V_b = 20 mV). NcAFM data was acquired with a CO-functionalised tip. **c,** Simulated vertical ncAFM map for a tri-iron node, reproducing the height dependence of the central feature **d, e, f, g** Simulated ncAFM images of the coordination node at different heights (indicated by dashed lines in **c**). See Methods in main text for details on the calculations. Scale bars: 5 Å.

These DFT-based ncAFM image simulations also reproduce our experimental ncAFM images for smaller values of *z*, where the central protrusion vanishes (see Supplementary Figs. 7c-g and 3). Indeed, while for values of $z \sim 0$ Å the frequency shift Δf at the central Fe is larger than at the surrounding ligands (resulting in the central protrusion in ncAFM imaging), the contrast is reversed when *z* decreases (e.g., $z < \sim -1$ Å), with Δf becoming smaller at the centre Fe than at the organic moiety, resulting in the vanishing of the protrusion seen in Supplementary Fig. 3.

Supplementary Note 3: (d*I*/d*V*)/(*I*/*V*) STS of metalated TPPT species

Supplementary Figure 8 shows (d*I*/d*V*)/(*I*/*V*) STS data for the metalated TPPT species resulting from the lateral STM manipulation in Fig. 3 of the main text. Spectra were determined by acquiring *I*(*V*) curves (with an initial STM setpoint I_t = 25 pA, V_b = -1 V) and calculating the numerical derivative. All data were acquired with the same Pt/Ir STM tip.

STS curves acquired at the centre of various TPPT species (inset Supplementary Fig. 8) show a pronounced feature at positive biases, associated with an empty molecular orbital.[11] The TPPT molecule, which each of its *tpy* groups coordinated to a single Fe adatom ($tpy\text{-}Fe^{(A)}$), shows a peak at 1.56 V. The species resulting from the deconstruction of the nanochain node ($tpy\text{-}Fe^{(D)}$) shows the same spectroscopic signature; both species are equivalent. The doubly metalated $tpy\text{-}Fe_2^{(A)}$ moiety, assembled lateral STM manipulation (see Fig. 3 of main text) exhibits a peak at 1.67 V (110 mV larger than the singly metalated species). The $tpy\text{-}Fe_2^{(D)}$ species, resulting from the deconstruction of the node, shows the same feature; $tpy\text{-}Fe_2^{(A)}$ and $tpy\text{-}Fe_2^{(D)}$ are equivalent.

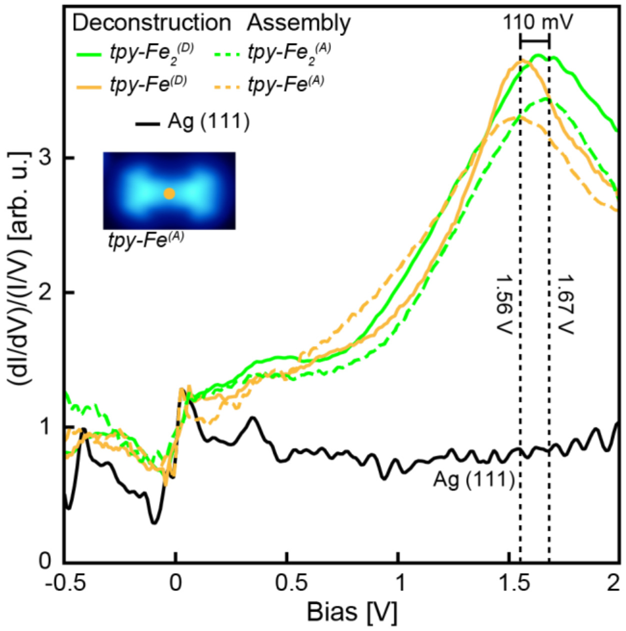

Supplementary Figure 8. (d*I*/d*V*)/(*I*/*V*) STS spectra for assembled species $tpy\text{-}Fe^{(A)}$, $tpy\text{-}Fe_2^{(A)}$ and those resulting from the *deconstruction* of the 1D chain node via lateral STM manipulation ($tpy\text{-}Fe^{(D)}$, $tpy\text{-}Fe_2^{(D)}$; see Fig. 3 of main text). *Tpy-Fe* (orange) consists of a *tpy* group metalated with a single Fe adatom. $Tpy\text{-}Fe_2$ (green) refers to a *tpy* interacting with 2 Fe adatoms. Spectra were acquired at the centre of the TPPT ligand [see STM image of Fe-TPPT-Fe in inset (I_t = 100 pA, V_b = -500 mV)]. Black curve: reference Ag(111) spectrum. Data extracted from (d*I*/d*V*)/(*I*/*V*) STS in Fig. 3 of main text.

Supplementary Note 4: Nature of the ncAFM central feature of the coordination node

Although CO-functionalised ncAFM allows for imaging of single chemical bonds within flat aromatic molecules, imaging of single metal atoms within a cluster or metal-organic complex is more challenging. Contrast has been achieved for specific metal-organic systems (e.g., complexes on insulators,[12] metallo-tetrapyrroles[13]), but only for metal atoms in the molecular plane.[9] Usually ncAFM imaging relies on changes of molecular conformation as indirect evidence for metal-organic coordination.[14,15,16] The central feature observed in our CO-tip ncAFM data for the coordination node can thus – *a priori* – have a number of possible explanations:

i. Interaction of Fe with residual gas molecules. Iron centres in metal-organic complexes are known for their high affinity to small gas molecules, e.g., oxygen[17], CO[1]. However, we have identified the effect on ncAFM imaging of the specific interaction between the Fe centre of our system and CO (see Supplementary Fig. 2 above). Moreover, our study was performed in UHV with an extremely low oxygen partial pressure ($p_{O2} < 1 \times 10^{-12}$ mbar), reducing the likelihood of interactions with O_2. We can hence rule out this hypothetical explanation.

ii. An Ag adatom from the substrate. Studies on Au(111)[16] and Cu(111)[15] have shown that reactive moieties, including *tpy*, can spontaneously form complexes with adatoms from the surface. This is not the case here, since the metalated structures only emerge after Fe deposition.

iii. A *cavity* effect. Let us assume a hypothetical scenario where there are no electrostatic interactions between the CO molecule and the tri-iron node in the MOC. That is, where the CO molecule is neutral, and tip-sample forces are of van der Waals (vdW) type. Now, let us consider the case where the ncAFM CO-tip is positioned above the node, at a height where the tip-sample vdW interactions are attractive. Since the facing *tpy*'s of the node form a *cavity* – with the central Fe lying closer to the surface than the molecular plane – the overall attractive interactions at the centre of the node can arguably be smaller than at its surrounding, resulting in an apparent saddle or protrusion, with a darker area around it (Supplementary Fig. 9d). Such a feature would be enhanced by Laplace filtering. Notably, this feature would also be present in scenarios which would not involve a central Fe atom (e.g., see Supplementary Fig. 10b). However, it is important to note that a central node protrusion due to this cavity effect would be a lot weaker in comparison to a protrusion given by electrostatic tip-sample interactions involving a partially charged CO (Supplementary Fig. 9). The latter is in better agreement with our experiments. Moreover, a central node protrusion solely due to this cavity effect would be inconsistent with our LCPD measurements.

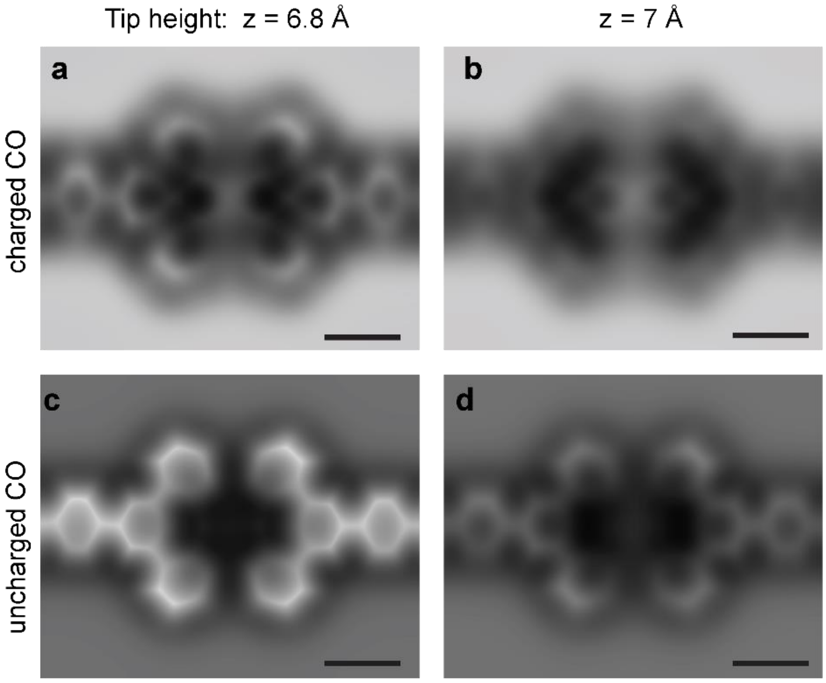

Supplementary Figure 9. Nature of the CO-tip ncAFM image protrusion at the centre of the tri-iron coordination node. a, b, DFT-simulated constant-height CO-tip ncAFM images of the tri-iron coordination node with electrostatic tip-sample interactions given by a partially negatively charged CO molecule[18] (quadrupole charge Q = -0.2 eÅ^2, that is, with negative lobe oriented towards sample), for different tip heights in the attractive regime. **c, d,** Same, without electrostatic forces (that is, neutral CO; Q = 0). The weak central node protrusion in (d) for an uncharged CO can be explained by the cavity effect (see above); its contrast is significantly smaller than for the charged case, which is in better agreement with our experiments. The experimentally observed central node protrusion can hence be interpreted as the result of a repulsive electrostatic interaction between partially charged CO and metal-organic coordination node. Scale bars: 5 Å.

Supplementary Note 5: Comparison with hypothetical di-iron coordination node

To corroborate our claim that the metal-organic coordination node consists of a tri-iron cluster, we also performed DFT calculations for a hypothetical scenario of a di-iron coordination node. The relaxed structure of the latter involves a similar flat head-to-head coordination motif. The node consists of two opposing *tpy* groups, each metalated with a single Fe adatom, with an axial N-N distance of 8.96 Å and an Fe-Fe distance of 5.56 Å. Supplementary Figure 10b shows a simulated CO-tip ncAFM image of this system. Compared to simulated ncAFM images of the tri-nuclear node (Supplementary Fig. 10a), the central protrusion is less sharp, with a significantly different height profile compared to the tri-nuclear node (Fig. 2 of main text), inconsistent with our experiments.

The calculated electrostatic potential of the di-iron node further strengthens this difference; the centre of the node exhibits a positive (that is, attractive) potential, in stark contrast to our LCPD experimental results which showed a strong negative electrostatic potential leading to a bright central feature in the CO-tip ncAFM imaging (Fig. 4 of main text). We can thus rule out the di-iron node as a possible configuration, from both the DFT calculations as well as from the STM manipulation experiments (Fig. 3 of main text).

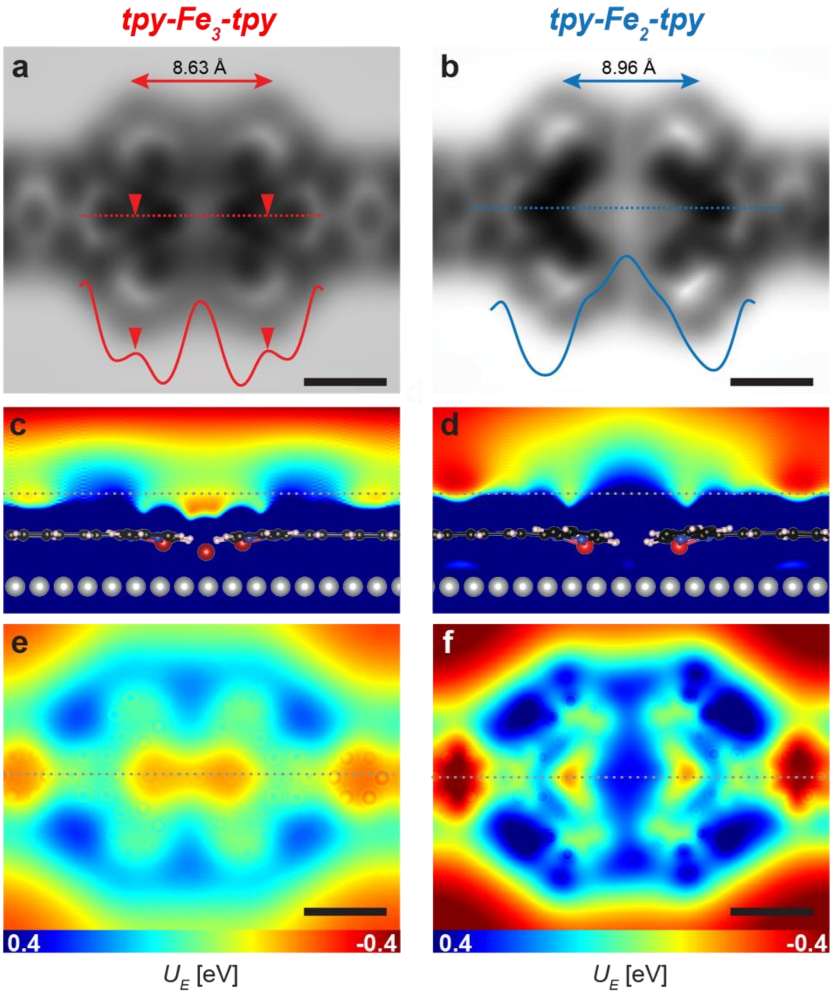

Supplementary Figure 10. Comparison between tri-iron and hypothetical di-iron coordination nodes. **a, b,** DFT-simulated CO-tip ncAFM images of a tri-iron and di-iron node, respectively. Apparent height line profiles across the centre of the nodes and distances between the axial pyr N atoms are indicated. **c, d, e, f**, DFT-simulated simulated electrostatic potentials for a tri- and di-iron node, respectively (vertical and horizontal slices along dashed lines). Scale bars: 5 Å.

We would like to emphasise the importance of our multi-technique approach to fully elucidate non-trivial metal-organic structures of unknown composition. CO-functionalised ncAFM imaging alone is not sufficient to unequivocally distinguish between the di-iron and tri-iron scenarios. In this study, LCPD and STM manipulation experiments provided key data to resolve the atomic-scale structure of the MOCs.

Supplementary Note 6: NEXAFS spectroscopy, Bader analysis and oxidation state of iron atoms

The concept of oxidation state of a metal atom in a metal-organic complex is useful in chemistry, materials science and condensed matter physics, since it is directly linked to its chemical reactivity. However, it is a classical concept that relies on the idea of an integer number of electrons per individual atom, and it is therefore ambiguously defined within the framework of quantum mechanics.[19, 20] In particular, it is not trivial to assign an oxidation state to an atom from first-principles calculations.[20]

To gain further insight into the chemical environment of the Fe atoms in the MOC trinuclear nodes, we performed Near-Edge X-ray Absorption Fine-Structure (NEXAFS) spectroscopy measurements.[21] These measurements were carried out at the Soft X-Ray Beamline of the Australian Synchrotron. Samples were prepared *in situ* following the same methods as for our STM and ncAFM measurements (see Methods in main text). NEXAFS spectra were acquired in partial electron yield (PEY) detection mode, averaging spectra acquired at multiple incidence angles with respect to normal (0°, 20°, 35°, 50°, 70°), with *p*-polarization, with the sample at room temperature. Supplementary Figure 11 shows Fe *L*-edge NEXAFS spectra for pure Fe (cyan) on Ag(111), and for Fe-TPPT (red) on Ag(111). The spectrum for Fe/Ag(111) shows two peaks L_{III} and L_{II} characteristic of neutral Fe, at photon energies of 706.7 and 718.75eV, corresponding to Fe $2p_{3/2} \rightarrow 3d$ and $2p_{1/2} \rightarrow 3d$ transitions, respectively.[22] For Fe-TPPT/Ag(111), in addition to the 706.7 eV peak, the spectrum shows a clear peak associated with the $2p_{3/2} \rightarrow 3d$ transition at a photon energy of 708.5 eV. A positive photon energy shift of this $2p_{3/2} \rightarrow 3d$ transition corresponds to an increase of related core level binding energy and hence to a positive change of the effective charge state of Fe. This 708.5 eV photon energy is very similar to that of the L_{III} peak for Fe in a 2+ oxidation state, observed for different Fe(II) oxide species.[23, 24, 25, 26] This 708.5 eV peak is a signature of the Fe-TPPT coordination, and indicates that the chemical environment of at least some of the Fe atoms involved in the coordination is similar to that of Fe in a 2+ oxidation state.

In parallel, we performed Bader charge analysis[27] for each individual Fe atom in the tri-iron coordination node, based on our DFT calculations. This Bader charge analysis yields a positive charge state for all three Fe atoms of the node, with the distal Fe (Bader charge ~ +0.8e) differing significantly from the central (~ +0.15e). Although this Bader analysis does not allow us to unambiguously determine the oxidation state of these Fe atoms, we can reliably claim that, given a Bader charge difference of ~0.65*e*, the electronic configurations (and hence chemical reactivity) of distal and central Fe's differ significantly. Indeed, previous DFT-based studies on metal-organic complexes[20] were able to associate Bader charge differences on the order of ±0.2*e* with differences in oxidation state on the order of ±1. This is a strong indication of a mixed valence configuration of the tri-nuclear coordination node.

Therefore, we attribute the 708.5 eV NEXAFS L_{III} peak in Supplementary Fig. 11 to the Fe atoms directly coordinated with TPPT; the chemical environment of the distal Fe in the tri-iron node is similar to that of Fe in a 2+ oxidation state. This is consistent with the Fe chemical environment observed in complexes composed of a single Fe(II) atom coordinated with polypyridyl ligands in a octahedral geometry.[28, 29, 30, 31, 32, 33]

Our Bader charge analysis yields a significantly less positive central Fe; the $2p_{3/2} \rightarrow 3d$ transition associated with the central Fe should thus occur at lower photon energies. The NEXAFS L_{III} peak at 706.7 for the red curve in Supplementary Fig. 11 indicates the presence of significant amounts of

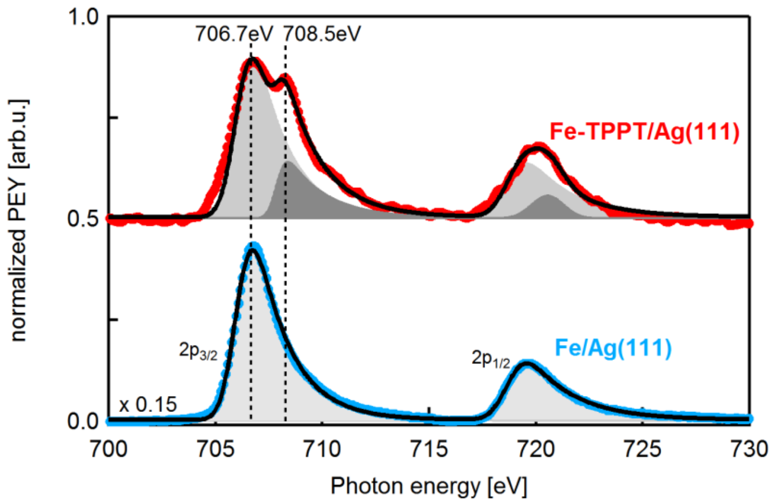

Supplementary Figure 11. Fe L-edge NEXAFS spectra of Fe (blue) on Ag(111) and Fe-TPPT (red) on Ag(111). When TPPT and Fe are co-deposited, a clear peak at 708.5 eV appears due to the Fe-TPPT coordination. Cyan curve: ~0.30 ML Fe; red: ~0.7 ML TPPT, ~0.01 ML Fe. Black curves represent total fits. Cyan (red) curve is fitted with two (four, respectively) asymmetric Gaussian functions, plotted as grey shaded areas. The spectra were acquired in partial electron yield (PEY) mode, with the sample at room temperature, and have been offset for clarity.

neutral Fe(0) in the Fe-TPPT/Ag(111) system. This can be explained by Fe adatoms attaching to Ag step edges or forming clusters, as observed by STM. The less positive central Fe atom in the MOC tri-iron nodes could also contribute to this peak. This would point to a chemical environment of the central Fe similar to that of neutral Fe(0). However, the low Fe coverage and 2:1 ratio between distal and central Fe atoms could result in a weak NEXAFS signal specifically related to the central Fe; we cannot rule out a chemical environment of the latter similar to that of Fe(I), in a 1+ oxidation state.

As stated above, assigning an integer oxidation state to a metal atom in a metal-organic system can be ambiguous in the framework of quantum mechanics.[19, 20] It is more meaningful to quantitatively characterize, if possible, the real-space atomic-scale electronic environment of the system. This would determine its chemical reactivity and functionality. Our combination of CO-tip ncAFM, LCPD mapping, DFT calculations, NEXAFS spectroscopy and Bader analysis allows us to unambiguously determine the atomic-scale electronic environment of each of the Fe atoms in a MOC coordination node. Importantly, our approach provides compelling evidence that the latter consists of two distal Fe atoms, each with a chemical environment close to that of Fe(II), and a central Fe with a chemical environment close to that of Fe(0) or Fe(I).

The DFT-calculated electrostatic potential in Fig. 4c of the main text is the result of a non-spherical charge distribution around the Fe atoms. Therefore, the point charge for each individual Fe atom resulting from our Bader analysis above may vary depending on the used projection (i.e., Bader, Mulliken,[34] Löwdin[35]). Our Bader analysis serves as a tentative approach to highlight the differences in chemical environment and valence state between distal and central Fe atoms. It is important to note that this DFT-derived Bader analysis is consistent with our NEXAFS measurements.

Our DFT calculations – which reproduce all of our ncAFM and LCPD experimental data – support a slightly kinked, v-shaped Fe trimer configuration, with the central Fe slightly closer to the surface in comparison to the two distal Fe (see Fig. 4f of main text). Therefore, the interaction between the substrate and the distal Fe atoms is arguably weaker than that for the central Fe (if there is any difference at all). This, in combination with the fact that the adsorption site of each of the Fe atoms in the trimer is quasi-identical [i.e., close to a Ag(111) hollow site; see main text], provides a strong indication that the significant difference in chemical environment between central and distal Fe atoms cannot be explained by a difference in adsorption site. It is a result of the metal-organic coordination.

Supplementary Note 7: Van der Waals parameters for AFM simulations

Simulated ncAFM images were obtained using the ProbeParticle code.[36] Within this model attraction due to London dispersion and Pauli repulsion are modelled by Lennard-Jones potentials:

$$E_{ij}(r_{ij}) = \epsilon_{ij}\left(\left(R_{ij}/r_{ij}\right)^{12} - 2\left(R_{ij}/r_{ij}\right)^{6}\right)$$

where ϵ_{ij} and R_{ij} are calculated using mixing rules from element-wise parameters (Lorentz-Berthelot):

$$\epsilon_{ij} = \sqrt{\epsilon_{ii}\epsilon_{jj}} \text{ and } R_{ij} = R_{ii} + R_{jj}.$$

The parameters used in the simulations are listed in the following table:

**Element**	$\boldsymbol{R_{ii}}$ **[Å]**	$\boldsymbol{\epsilon_{ii}}$ **[meV]**
**H**	1.4870	0.681
**C**	1.9080	3.729
**N**	1.7800	7.370
**O**	1.6612	9.106
**Ag**	2.3700	10.000
**Fe**	2.0000	10.000

Supplementary Table 2. Van der Waals parameters used in the ncAFM simulations. Note that reasonable parameter values for organic elements are well known [from Optimized Potentials for Liquid Simulations (OPLS)[37, 38]], while precise values for metals (Ag, Fe) do not significantly impact the observed ncAFM contrast.

Supplementary Note 8: Chain adsorption geometry

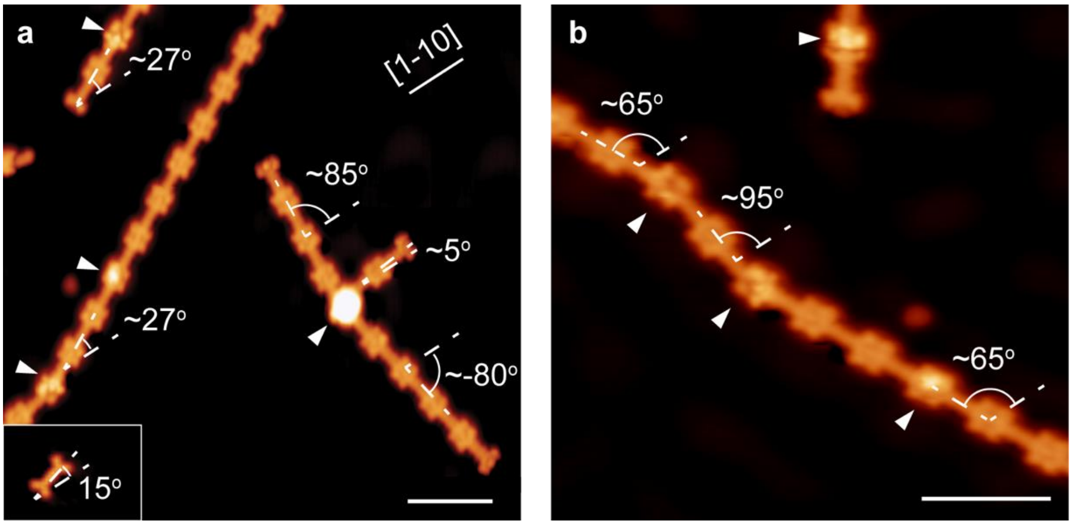

Supplementary Figure 12. a, b, Constant-current STM topography images of MOCs on Ag(111) (It = 25 pA, Vb = 20 mV). The STM tip was functionalised by picking up a single carbon monoxide molecule (Methods). Angles between the MOCs and Ag [1-10] direction are indicated. The orientation of the TPPT molecules within the MOCs with respect to the substrate crystalline axes is altered in comparison with an isolated molecule (in inset). Note that several nodes are decorated or contain an Fe cluster (white arrows). Scale bars: 5 nm.

Supplementary Figure 12 shows constant-current STM images of MOCs on Ag(111). In contrast to single TPPT molecules (inset in Supplementary Fig. 12), which align exclusively at ± ~15° with respect to the substrate crystalline axes, the MOCs exhibit additional orientations (± 5°, 20°, 27°, 35°); there is no specific orientation of the MOCs with respect to the surface crystalline axes. That is, the influence of the surface on the MOC growth orientation is smaller than on the pristine TPPT adsorption orientation. This is a clear indication that the chaining reduces the interaction between surface and molecule. Importantly, the tri-iron coordination nodes in the MOCs are imaged identically by STM independently of the MOC orientation. The fact that we observe different MOC orientations – as well as the fact that the latter do not influence the STM imaging of the tri-iron nodes – shows that the coordination geometry is dominated by the Fe-Fe and Fe-TPPT interaction rather than by interactions with the substrate.

Supplementary References

1. Seufert K., Auwärter W., Barth J.V. Discriminative response of surface-sonfined metalloporphyrin molecules to carbon and nitrogen monoxide. *J. Am. Chem. Soc.* **132**, 18141-18146 (2010).
2. Kulawik M., et al. Interaction of CO molecules with surface state electrons on Ag(111). *Surface Science* **590**, L253-L258 (2005).
3. Hapala P., Kichin G., Wagner C., Tautz F.S., Temirov R., Jelínek P. Mechanism of high-resolution STM/AFM imaging with functionalized tips. *Phys Rev B Condens Matter* **90**, 085421 (2014).
4. Gross L., Mohn F., Liljeroth P., Repp J., Giessibl F.J., Meyer G. Measuring the charge state of an adatom with noncontact atomic force microscopy. *Science* **324**, 1428-1431 (2009).
5. Gross L., *et al.* Bond-order discrimination by atomic force microscopy. *Science* **337**, 1326-1329 (2012).
6. Hanssen K.Ø., et al. A combined atomic force microscopy and computational approach for the structural elucidation of breitfussin A and B: Highly Modified Halogenated Dipeptides from Thuiaria breitfussi. *Angewandte Chemie International Edition* **51**, 12238-12241 (2012).
7. Körzdörfer T., Brédas J.-L. Organic electronic materials: recent advances in the DFT description of the ground and excited states using tuned range-separated hybrid functionals. *Accounts of Chemical Research* **47**, 3284-3291 (2014).
8. Tershansy M.A., Goforth A.M., Peterson Jr L., Burns M.C., Smith M.D., zur Loye H.-C. Syntheses and crystal structures of new chain-containing iodometallate compounds: [H1,10-phen]$(H_2O)_{1.41}[AgI_2]$, [H1,10-phen]$(H_2O)_{1.42}[CuI_2]$; [Co(tpy)$_2$][Bi_2I_8], [Fe(tpy)$_2$][Bi_2I_8]; [Co(1,10-phen)$_3$][Pb_3I_8]·H_2O, and [Fe(1,10-phen)$_3$][Pb_3I_8]·0.5(H_2O). *Solid State Sciences* **9**, 895-906 (2007).
9. Albrecht F., Bischoff F., Auwärter W., Barth J.V., Repp J. Direct identification and determination of conformational response in adsorbed individual non-planar molecular species using non-contact atomic force microscopy. *Nano Lett.* **16**, 7703–7709 (2016).
10. Mohn F., Gross L., Meyer G. Measuring the short-range force field above a single molecule with atomic resolution. *Appl. Phys. Lett.* **99**, 053106 (2011).
11. Capsoni M., et al. Selective hybridization of a terpyridine-based molecule with a noble metal. *The Journal of Physical Chemistry C* **121**, 23574-23581 (2017).
12. Albrecht F., Neu M., Quest C., Swart I., Repp J. Formation and characterization of a molecule–metal–molecule bridge in real space. *J. Am. Chem. Soc.* **135**, 9200-9203 (2013).
13. He Y., et al. Fusing tetrapyrroles to graphene edges by surface-assisted covalent coupling. *Nat. Chem.* **9**, 33-38 (2016).
14. Kocić N., et al. Control of reactivity and regioselectivity for on-surface dehydrogenative aryl–aryl bond formation. *J. Am. Chem. Soc.* **138**, 5585-5593 (2016).
15. Zint S., et al. Imaging successive intermediate states of the on-surface Ullmann reaction on Cu(111): role of the metal coordination. *ACS Nano* **11**, 4183-4190 (2017).
16. Pawlak R., et al. Design and characterization of an electrically powered single molecule on gold. *ACS Nano* **11**, 9930–9940 (2017).
17. Fabris S., et al. Oxygen dissociation by concerted action of di-iron centers in metal-organic coordination networks at surfaces: modeling non-heme iron enzymes. *Nano Lett* **11**, 5414-5420 (2011).
18. Peng J., et al. Weakly perturbative imaging of interfacial water with submolecular resolution by atomic force microscopy. *Nat. Commun.* **9**, 122 (2018).
19. Raebiger H., Lany S., Zunger A. Charge self-regulation upon changing the oxidation state of transition metals in insulators. *Nature* **453**, 763-766 (2008).
20. Reeves K.G., Kanai Y. Theoretical oxidation state analysis of Ru-(bpy)$_3$: Influence of water solvation and Hubbard correction in first-principles calculations. *J. Chem. Phys.* **141**, 024305 (2014).

21. Stohr J. *NEXAFS Spectroscopy*. Springer (1992).

22. Tobin J.G., Waddill G.D., Pappas D.P. Giant X-ray absorption circular-dichroism in magnetic ultrathin films of Fe/Cu(001). *Phys. Rev. Lett.* **68**, 3642-3645 (1992).

23. Park T.J., Sambasivan S., Fischer D.A., Yoon W.S., Misewich J.A., Wong S.S. Electronic structure and chemistry of iron-based metal oxide nanostructured materials: a NEXAFS investigation of $BiFeO_3$, $Bi_2Fe_4O_9$, alpha-Fe_2O_3, gamma-Fe_2O_3, and Fe/Fe_3O_4. *J. Phys. Chem. C* **112**, 10359-10369 (2008).

24. Zheng F., Perez-Dieste V., McChesney J.L., Luk Y.Y., Abbott N.L., Himpsel F.J. Detection and switching of the oxidation state of Fe in a self-assembled monolayer. *Surface Science* **587**, L191-L196 (2005).

25. Otero E., Wilks R.G., Regier T., Blyth R.I.R., Moewes A., Urquhart S.G. Substituent effects in the iron 2p and carbon 1s edge near-edge X-ray absorption fine structure (NEXAFS) spectroscopy of ferrocene compounds. *J. Phys. Chem. A* **112**, 624-634 (2008).

26. Otero E., Kosugi N., Urquhart S.G. Strong double excitation and open-shell features in the near-edge X-ray absorption fine structure spectroscopy of ferrocene and ferrocenium compounds. *J. Chem. Phys.* **131**, 114313 (2009).

27. Tang W., Sanville E., Henkelman G. A grid-based Bader analysis algorithm without lattice bias. *J. Phys.: Condens. Matter* **21**, 084204 (2009).

28. Brown A.M., McCusker C.E., McCusker J.K. Spectroelectrochemical identification of charge-transfer excited states in transition metal-based polypyridyl complexes. *Dalton Trans.* **43**, 17635-17646 (2014).

29. Bernhard S., Goldsmith J.I., Takada K., Abruna H.D. Iron(II) and copper(I) coordination polymers: Electrochromic materials with and without chiroptical properties. *Inorg. Chem.* **42**, 4389-4393 (2003).

30. Bowman D.N., Blew J.H., Tsuchiya T., Jakubikova E. Elucidating band-selective sensitization in iron(II) polypyridine-TiO_2 Assemblies. *Inorg Chem* **52**, 8621-8628 (2013).

31. Liu B., Huang H.X., Zhang C.F., Chen M., Qian D.J. Monolayers, Langmuir-Blodgett films of bimetallic coordination polymers of 4 '-(4-pyridyl)-2,2 ': 6 ',2 "-terpyridine. *Thin Solid Films* **516**, 2144-2150 (2008).

32. Johnson P.S., et al. Electronic structure of Fe- vs. Ru-based dye molecules. *J. Chem. Phys.* **138**, 044709 (2013).

33. Johansson E.M.J., et al. Spin-orbit coupling and metal-ligand interactions in Fe(II), Ru(II), and Os(II) Complexes. *J. Phys. Chem. C* **114**, 10314-10322 (2010).

34. Mulliken R.S. Electronic population analysis on LCAO-MO molecular wave functions. I. *J Chem Phys* **23**, 1833-1840 (1955).

35. Lowdin P.O. On the non-orthogonality problem connected with the use of atomic wave functions in the theory of molecules and crystals. *J. Chem. Phys.* **18**, 365-375 (1950).

36. Hapala P., et al. Mapping the electrostatic force field of single molecules from high-resolution scanning probe images. *Nat. Commun* **7**, 11560 (2016).

37. Jorgensen W.L., Tirado-Rives J. The OPLS [optimized potentials for liquid simulations] potential functions for proteins, energy minimizations for crystals of cyclic peptides and crambin. *J. Am. Chem. Soc.* **110**, 1657-1666 (1988).

38. Jorgensen W.L., Maxwell D.S, Tirado-Rives J. Development and testing of the OPLS all-atom force field on conformational energetics and properties of organic liquids. *J. Am. Chem. Soc.* **118**, 11225-11236 (1996).
